# Yes

**Published:** 1923-02

**Authors:** Arthur Stanley

**Affiliations:** G.B.E., (Treasurer of St. Thomas's Hospital)


### THE COMBINED APPEAL: HAS IT BEEN SUCCESSFUL?

**YES.—By the HON. SIR ARTHUR STANLEY, G.B.E. (Treasurer of St. Thomas's Hospital).**

THE Combined Hospitals Appeal for London has ended with the outgoing year, and although the final efforts are not complete it is possible to form at least some first conclusions upon the experiment as a whole. It was, of course, a new departure, and it is useful to consider its value so far as the finances of the hospitals are concerned and its importance in deciding the general direction of future hospital policy.

### The Appeal Successful.

It will at once be agreed that the appeal has been successful. Mr. Shrapnell Smith and his staff set out to raise £500,000, and we know from the estimate that has been given to us—presumably conservative—that that amount is quite, or at all events, very nearly, within sight. What the cost has been has not yet been made public. It is certainly not excessive, and as it is met out of moneys provided by King Edward's Fund it is not a matter of great importance to the hospitals themselves. What does matter to them is that a sum of half a million pounds has been raised within the last twelve months to assist in lightening the load of debt that has been hanging round their necks since the early years of the war.

### Its Origin.

The origin of the appeal must be remembered. When hospitals, not only in London but throughout the country, found themselves in difficulties some five or six years ago, owing to the heavy cost of renewals and repairs, the phenomenal rise in prices and wages and the shrinkage of subscriptions, they turned to the Government for assistance. The Commission known as Lord Cave's was appointed, and after careful examination of the whole subject produced a very valuable report. Excellent recommendations were made, but of these the only one that interests us at the moment is the recommendation that £1,000,000 a year for two years should be granted to the voluntary hospitals, in order to enable them to set their house in order, to relieve them from their temporary embarrassment, and to give them breathing space during which they could spread wide their nets to catch the vast number of small subscriptions which they had hitherto not required, owing to the comparative ease with which they could obtain the necessary funds from the rich. There can be no doubt that had this recommendation been adopted by the Government the grant of the sum of £2,000,000 would have achieved its object, and there would have been no need for this Combined Appeal. But for reasons which are well known to everyone the Government did not see its way to give the sum recommended, and in its place provided only £500,000 spread over two years. Whether the Government was wise or not in coming to this decision it is not for us to inquire. We all know how many are the calls upon the public purse, and we are painfully conscious of the difficulty found in filling it. One thing was certain—a great voluntary effort was necessary to supply the place of the Government grant which was lacking. King Edward's Hospital Fund for London took the matter up and fathered the Combined Appeal.

### A Question of Distribution.

In the brief space of this article it is only possible to deal with two aspects of this appeal—was it successful and how far has it affected the voluntary hospitals of London and their future policy? The first question has already been answered. The Appeal has produced the sum which it set out to obtain; one half of the total £500,000 has already been distributed; the other half will, it is hoped, shortly be divided among the participating hospitals. The trustees of the fund have, in their first distribution, mainly proceeded on the lines of making grants in proportion to the respective deficits of individual hospitals incurred in the year 1921. It is to be hoped that, in the distribution of the second half of the fund, consideration will be given to the efficiency of the hospital receiving a grant, and to the results that it is achieving. There is every reason to believe that those responsible for the distribution have these most important points in mind.

### Future Policy.

The second question is more difficult to answer, as it is a matter of opinion and not of fact. When the idea of a Combined Appeal was first mooted it was only natural that those who were responsible for raising money, on the old lines, for individual hospitals should feel some fear lest this new form of appeal should interfere with their existing methods, and should permanently damage the sources from which, for years past, they had been in the habit of drawing the funds necessary for the maintenance of their particular hospitals. Whether the Combined Appeal has had any such effect either in a small or large degree it is impossible to estimate at present. I have heard the representative of one of the largest hospitals state publicly that his hospital had lost £20,000 owing to the action of the Combined Appeal, but I should be curious to hear his reasons for this opinion, and I am certain that the other large hospitals would tell quite a different tale. Moreover, in the case of the hospital mentioned above, if there is a loss, it tends to show that it was encroaching on the ground of others and getting more than its fair share of the money subscribed by the general public. Still, it is possible that where many hospitals have undoubtedly gained by the Combined Appeal, some few may have lost.

### The Gain of Co-ordination.

On the other side of the account we have the undoubted gain accruing from the co-ordination and concentration of effort. Out of some 110 voluntary hospitals in London it is safe to say that at least 70 were making appeals, and each one of those appeals was costing money and competing with appeals made by other hospitals. The amount saved, not only in cash but in waste, due to overlapping, is not a calculable quantity but must certainly be large. Another great gain is the quickening of the interest of the general public of London in their hospitals and the awakening of the local patriotism of London as a whole. Throughout the United Kingdom there is in practically every county a county hospital, or some central institution that does duty as such. Whenever such a hospital is in need of funds it appeals to the local patriotism of the county, and it is seldom that the appeal fails. Such a sentiment did not exist in London, except in so far as it was present in a rather negative form in King Edward's Fund, which Fund was principally engaged, until quite lately, in receiving money rather than collecting it, and in exercising a general supervision over the interior economy of individual hospitals, rather than providing for the co-ordination of their work with a view to the systematic treatment of the sick poor.

### London Keen for Preservation.

The fact that this local patriotism has been aroused, and that Londoners are not only keen upon the preservation of their hospitals, but are proud of them and of the work they do, is, to my mind, almost as great an achievement as the actual raising of so large a sum as £500,000. For the manner in which the appeal has been carried out, for the vigour and efficiency of the campaign, the highest praise is due to Mr. Shrapnell Smith and his able and zealous band of workers. The grateful thanks of all the London hospitals are also due to King Edward's Fund, and to those who direct its policy, for the manner in which they have met a great emergency, and gone far towards solving the problem of the maintenance of the voluntary system to which the country has owed so much in the past and on which so largely depends the health and well-being of future generations.

